# The relationships between *ACTN3* rs1815739 and *PPARA-α* rs4253778 gene polymorphisms and athletic performance characteristics in professional soccer players

**DOI:** 10.1186/s13102-023-00733-0

**Published:** 2023-09-25

**Authors:** Celal Bulgay, Ladislav Cepicka, Metin Dalip, Selin Yıldırım, Halil İ. Ceylan, Özlem Ö. Yılmaz, Korkut Ulucan, Georgian Badicu, Mesut Cerit

**Affiliations:** 1 Sports Science Faculty, Bingol University, Bingöl, 12000 Türkiye; 2 Department of Physical Education and Sport, Faculty of Education, University of West Bohemia, Pilsen, 30100 Czech Republic; 3 Faculty of Physical Culture and Health, University in Tetovo, Tetova, 1200 Republic of North Macedonia; 4 Sports Science Faculty, Lokman Hekim University, Ankara, 06510 Türkiye; 5 Kazim Karabekir Faculty of Education, Ataturk University, Erzurum, 25240 Türkiye; 6 Institute of Health Sciences Marmara University, İstanbul, 34722 Türkiye; 7 Department of Medical Biology and Genetics, Marmara University, İstanbul, 34722 Türkiye; 8 Faculty of Physical Education and Mountain Sports, Transilvania University of Braşov, Brasov, 500068 Romania

**Keywords:** *ACTN3* rs1815739, Athletic performance, Polymorphisms, *PPARA-α* rs4253778, Soccer

## Abstract

**Background:**

Current research on athletic performance focuses on genetic variants that contribute significantly to individuals’ performance. *ACTN3* rs1815739 and *PPARA-α* rs4253778 gene polymorphisms are variants frequently associated with athletic performance among different populations. However, there is limited research examining the pre-and post-test results of some variants of athletic performance in soccer players. Therefore, the presented research is to examine the relationships between the *ACTN3* rs1815739 and PPARA-α rs4253778 gene polymorphisms and athletic performance improvement rates in adaptations to six weeks of training in elite soccer players using some athletic performance tests.

**Methodology:**

Twenty-two soccer players between the ages of 18 and 35 voluntarily participated in the study. All participants were actively engaged in a rigorous six-day-a-week training program during the pre-season preparation period. Preceding and following the training program, a battery of diverse athletic performance tests was administered to the participants. Moreover, Genomic DNA was extracted from oral epithelial cells using the Invitrogen DNA isolation kit (Invitrogen, USA), following the manufacturer’s protocol. Genotyping was conducted using real-time PCR. To assess the pre- and post-test performance differences of soccer players, the Wilcoxon Signed Rank test was employed.

**Results:**

Upon analyzing the results of the soccer players based on the *ACTN3* genotype variable, it was observed that there were no statistically significant differences in the SJ (Squat Jump), 30m sprint, CMJ (Counter Movement Jump), and DJ (Drop Jump) performance tests (*p > 0.05*). However, a statistically significant difference was identified in the YOYO IRT 2 (Yo-Yo Intermittent Recovery Test Level 2) and 1RM (One Repetition Maximum) test outcomes (YOYO IRT 2: CC, CT, and TT, *p* = 0.028, 0.028, 0.008, 0.000, respectively; 1RM: CC, CT, and TT, *p* = 0.010, 0.34, 0.001, respectively). Regarding the *PPARA-α* genotype variable, the statistical analysis revealed no significant differences in the SJ, 30m sprint, CMJ, and DJ performance tests (*p* > 0.05). Nevertheless, a statistically significant difference was observed in the YOYO IRT 2 and 1RM test results (YOYO IRT 2: CC, CG *p* = 0.001, 0.020; 1RM: CC, *p* = 0.000)

**Conclusions:**

The current study demonstrated significant enhancements in only YOYO INT 2 and 1RM test outcomes across nearly all gene variants following the six-day-a-week training program. Other performance tests, such as the 30m sprint, SJ, CMJ, and DJ tests did not exhibit statistically significant differences. These findings contribute novel insights into the molecular processes involving *PPARA-α* rs4253778 and *ACTN3* rs1815739 that underpin enhancements in endurance (YOYO INT 2) and maximal strength (1RM) aspects of athletic performance. However, to comprehensively elucidate the mechanisms responsible for the association between these polymorphisms and athletic performance, further investigations are warranted. It is thought that the use of field and genetic analyses together to support each other will be an important detail for athletes to reach high performance.

## Background

Sports scientists and coaches have observed that there are significant differences in physical performance improvement among individuals and that exercise loads contribute significantly to performance improvement by triggering individual behavioral changes. Understanding how and why genetic diversity affects behavioral changes in response to stimuli is crucial. With the completion of the Human Genome Project in 2003, there was a significant increase in the number of genetic analyses due to the technological acceleration in genetic analysis methods. Genetic research is now focused on studies that involve multiple genes rather than just one gene region. These studies examine and interpret the entirety of the genetic information (genome) transmitted to the organism, as well as the multi-factorial processes that encode proteins [1].

The degree of physical activity influenced by genetic variations can be determined by various factors. The presence or absence of a nucleotide in a specific region of a gene, for any reason, can make individual differences apparent. Among the most important factors contributing to the success of top athletes are environmental adaptation, optimal training loads, and random genetic sequence matches [2–4]. In addition, individual differences in exercise habits resulting from phenotypic changes related to inheritance are attributed to familial effects. Research on genetic markers related to the emergence of physical ability or talent has observed that individuals respond differently to acute and chronic exercise [2, 5]. It is widely accepted that athletic performance improvement has a very high genetic component for complex traits such as endurance, muscle strength, power, speed, agility, recovery rate, and risk of injury [6]. It is indisputable that genetic diversity affects both exercise performance and adaptation [7, 8]. Elite athletes can compete at the highest level due to the advantages of correct genetic marker matches. In addition to environmental factors, lifestyle choices, and motivation, the combined effect of multiple factors, such as the proper sequencing of inherited traits, facilitates reaching the highest level of athletic performance [9]. Indeed, numerous studies to date have found significant relationships between the Angiotension Converting Enzyme (*ACE*) and α-actinin-3 (*ACTN3*) genes and athletic performance. Similarly, it is well-known that the Peroxisome Proliferator-Activated Receptor Alpha (*PPARA-α*) gene is highly influential in the development of physical performance.

In the context of the development of athletic performance, the *ACTN3* gene, which is the most extensively researched, is associated with the production of the α-actinin-3 protein that plays structural and regulatory roles in muscle contraction [10–12]. Alpha-actinins contribute to producing more power and high-speed strength by activating fast-twitch muscle fibers during explosive activities that require speed and strength [4, 13]. The presence of the *ACTN3* gene in skeletal muscle activity is associated with superpower and exceptional endurance performance [14]. *ACTN3*, the main gene of the Z line that controls muscle contraction intensity, is also responsible for the production of the actin-binding protein alpha actin-3 [15, 16]. The *ACTN3* protein can cross-link with the cells of fast-twitch muscle fibers through thin actin filaments. The *ACTN3* gene polymorphism is determined by the presence of X or R at the R577 position. The R577X polymorphism (rs1815739 of the gene) codes for alpha-actinin-3, which substitutes an arginine residue at codon 577 instead of an early stop codon, resulting from the substitution of the "C" base at position 1,747 in exon 16 with a "T" base. Most importantly, this polymorphism triggers the production capacity of strong muscle contractions [14, 17, 18]. It is believed that the alpha-actinin-3 deficiency TT polymorphism hinders elite athletic performance in power and sprint sports (sprint, jump, and throwing events) [12, 19, 20].

Another important gene that triggers the provision of energy sources needed for long-term efforts through carbohydrates and fats is the peroxisome proliferator-activated receptor alpha (*PPARA-α*). The *PPARA-α* rs4253778 polymorphism located on the 22nd chromosome (22q12-q13.1) is associated with aerobic endurance and strength. *PPARA-α* is a molecule that contributes to the occurrence of metabolic variations depending on various nutritional conditions, especially related to carbohydrate, lipid, and amino acid metabolism [21]. Furthermore, the *PPARA* gene, which supports fatty acid activation, has been associated with the utilization of fatty acids, especially in the heart and skeletal muscles. The *PPARA-α* transcription factor regulates not only lipid, glucose, and energy balance but also body weight and vascular inflammation. While *PPARA* expression is observed at low levels in various tissues such as the pancreas, it is found at high levels in tissues that catabolize fatty acids, including the liver, skeletal muscle, and heart.

Type I (slow-twitch) muscle fibers have higher levels of PPARA-α expression (functional protein production) compared to type II (fast-twitch) muscle fibers. In addition to regulating the expression of a number of important muscle enzymes involved in fatty acid oxidation [22], *PPARA-α* also plays a significant role in the development and adaptation of aerobic endurance capacity [21, 23]. The most frequently analyzed genetic variant of the *PPARA* gene is the G/C polymorphism (rs4253778). In activities that require long-term effort where aerobic capacity is dominant, the GG genotype is associated with increased fatty acid oxidation in skeletal muscles and it has been observed that this genotype is more advantageous compared to others. It has been found that individuals with the GG genotype, who efficiently use the amount of oxygen sent to the tissue in order to maintain the continuity of muscle activity during long-term and low-intensity (more than 30 minutes) physical activities, also have higher proportions of slow-twitch type I muscle fibers [21]. While prolonged contraction times demonstrated by slow-twitch type I muscle fibers in athletes engaged in long-term and low-intensity physical activities contribute positively to performance development [24–26], carriers of the C allele with a high proportion of fast-twitch muscle fibers have been reported to exhibit better anaerobic capacity performance in speed and power-focused activities [27].

In literature, there are currently approximately 251 gene polymorphisms have been associated with athlete status, of which 128 genetic markers were positively associated with athlete status in at least two studies. Among these, 41 have been reported to be associated with endurance performance, 45 with power, and 42 with strength performance [28]. However, it is certain that more polymorphisms are needed to predict the physical performance level at the elite level for the candidate genes that affect physical performance [29]. Given the information described above, the aim of the present study is to investigate the relationships between the *ACTN3* rs1815739 and *PPARA-α* rs4253778 gene polymorphisms and physical performance characteristics in professional soccer players.

## Material and methods

### Participants

The current study included professional soccer team players (n = 22; mean age ± SD:24.79 ± 4.56; height (cm): 180.62 ± 4.88; body weight (kg): 74.42 ± 5.42) from the North Macedonia Super League. At the University of Tetova in North Macedonia, written informed consent forms containing all information such as the study protocol and results, were signed by the athletes before the study, and then an oral DNA sample was collected from each soccer player using a cotton swab for genetic analysis. Tetowa University Ethics Committee approved the study protocol (18.05.2022/02-1474/1) and the study procedure was in accordance with the principles of the Helsinki (II) Declaration. A six-week exercise program was applied to all soccer players. The physical performance characteristics (CMJ, DJ, YOYO IRT 2 test, 1 RM test, 30 m sprint test) of the players were measured before and after the exercise program.

### Exercise program

All participants were involved in a six-day-a-week training program (including aerobic and anaerobic exercise activities; Table 1), ranging from 35–150 minutes per session. The training program has a weekly volume of 13 sessions. The program consists of two microcycles per week, with three training sessions per microcycle and two exercise sessions per day (morning and evening) approximately totaling 900 minutes per week. The program includes exercises to develop anaerobic and aerobic capacity, intermuscular and intramuscular coordination, speed, and plyometric training loads.

**Table 1:** Training program applied for 1 microcycle in the pre-season preparation period

Week 1	Monday	Tuesday	Wednesday	Thursday	Friday		Saturday	Sunday
Morning	Strength Training Session	Coordination and Basic plyometric	Strength Training Session	Regeneration		Strength Training Session	Coordination Basic and plyometric	Strength Training Session
Evening	Soccer training + Aerobic Endurance Training	Soccer training + Aerobic Endurance Training	Preparation of Match/Compensation Training for nonstarters and less-minuted players	Free	Soccer training + Aerobic Endurance Training		Soccer training + Aerobic Endurance Training	Preparation of Match/Compensation Training for nonstarters and less-minuted players
Volume	65 + 85 min.	75 + 70 min.	40 + 90 min.	35–40 min.	65 + 85 min.		75 + 70 min.	40 + 90 min.
Intensity	Moderate-High	High	Moderate-High	Low	Moderate-High		High	Moderate-High
Metabolic	75–90% MHR	% 95 MHR	75–90% MHR	50–60% MHR	75–90% MHR		% 95 MHR	75–90% MHR
Mechanical	< 5000–6000 m	6000–7000 m	9000–13000 m	1000–2000 m	< 5000–6000 m		6000–7000 m	9000–13000 m

MHR: Maximum Heart Rate

### Procedures

All the soccer players who participated in the study were familiar with the testing procedures beforehand due to the training and performance evaluation practices at their clubs. The same test protocols were performed for the pre-and post-tests. Before the tests, the athletes completed traditional warm-up exercises such as general exercises (5 minutes of running at moderate speed followed by 3 minutes of active lower extremity stretching) and specific exercises (up to six maximum attempts at the exercises being tested). Players were given three minutes of rest after warming up before performing the tests. Jump tests were conducted using Optojump equipment in the study. Participants repeated the jumping tests five times. the Squat Jump (SJ) test is a widely employed method to assess an athlete’s explosive lower-body power, focusing on their muscle strength and power capabilities. The Counter Movement Jump Test (CMJT) is utilized to gather valuable insights into an athlete’s explosive leg strength. On the other hand, the Drop Jump Test is a valuable tool for obtaining information about an athlete’s reactive explosive strength, which involves the ability to generate force rapidly during a quick change of direction [30]. Data concerning the players’ recovery skills was acquired through the utilization of the YOYO Intermittent Recovery 2 test. This test is specifically designed to evaluate an athlete’s ability to recover and perform during intermittent high-intensity activities, providing valuable information about their endurance and recovery capacities in demanding sports scenarios.. In addition, data on the One Repetition Maximum (1RM) test (half squat) and the 30-meter sprint test were recorded using the Witty Photocell device.

### Data collections

#### Countermovement jump test (CMJ)

In the present study, SJ and CMJ tests were used to measure participants’ vertical jumping capacity. Before the jump attempt in SJ, the legs were held in a static position at 90° knee flexion angle for 2 seconds without any warm-up exercises. To prevent variations in the jumping coordination pattern during CMJ, the athletes were instructed to extend their lower extremities fully upward immediately after the downward movement, and all jumps were performed with hands on hips. Each jump was attempted five times at 15-second intervals using an Infrared Contact Platform (Opto Jump; Microgate, Italy) to measure flight time (t), which was used to calculate the distance (h) that the body’s center of mass traveled during the jump (h = gt^2^/ 8 ve g = 9,81 ms^2^). The takeoff and landing positions of a specific jump were considered valid for the study if they were visually comparable. The best-effort record was used for data analysis [30].

#### Drop jump (DJ) test

In the current study, the DJ performance was carried out using the Opto Jump Infrared Contact Platform from Microgate, Italy. The DJ was used to measure the athlete’s reactive explosive power. The DJ test involves athletes standing on a platform behind power plates, stepping forward onto the plates, and explosively jumping vertically immediately after the drop (during tests, the athletes’ hands are in contact with their hips). When the athlete lands on the platform, they quickly transition from the eccentric contraction phase to the amortization and concentric contraction phase. In this way, the device measures jumping performance [30].

#### YOYO intermittent recovery 2 (YOYO IRT 2) test

In the last decade, numerous studies have been conducted on the physical requirements and exercise profiles of several sports that involve intermittent activities such as basketball and soccer [31–33]. These types of sports require a lot of physical effort due to the many rapid and intense movements, including jumps, turns, battles, high-intensity sprints, and runs [34, 35]. Physiological findings from previous studies have shown that many of these sports have a high aerobic and anaerobic energy metabolism during competitions [36, 37]. The YOYO IRT 2 test is similar to the Leger shuttle-run test in that it consists of 20-meter shuttle runs with a rest period between each run. However, the YOYO IRT 2 test consists of two sets of shuttle runs at increasing speeds, separated by 10-second active recovery periods (controlled by audio signals from a compact disk player). The distance reached at the moment of exhaustion determines the test result. Participants continue to run until they can no longer maintain the current speed. The YOYO IRT 2 test lasts 5–15 minutes measures and the ability of a trained individual to perform repeated high-intensity exercises with a high anaerobic energy contribution [38, 39].

#### One repetition maximum test (1RM) protocol (Half Squat)

The one-repetition maximum (1RM) test is often considered the “gold standard” for evaluating a person’s strength capacity outside of the laboratory. Simply put, it can be defined as the maximum weight that a person can lift for only one repetition using proper technique. The purpose of the test is to determine the maximum weight that can be lifted for a complete repetition of an exercise, based on the assessment of different muscle groups.

The basic procedures applied for the 1RM (or any multiple RM) test are summarized below:

1. The participant warms up by performing several submaximal repetitions.
2. With three to five-minute rest intervals between each attempt, four attempts are made to determine the 1RM (or any multiple RM).
3. An initial weight is selected for the participant between 50–70% of their perceived capacity.

The weight resistance is gradually increased by 2.5 kg increments up to 20 kg, until the participant can no longer complete the chosen repetition. To ensure consistency between attempts, each repetition is performed at the same movement speed and range of motion. Therefore, the protocol mentioned above was used to determine the 1RM of soccer players [40].

#### 30-m sprint test

The 30-m sprint performance was measured using the Witty Speed, Microgate Equipment, and ITA device. The soccer l players, who were standing 0.5m away from the starting line, performed two sprint runs. The sprint tests were conducted on an indoor running track to prevent the effects of weather conditions. The distance covered during a specific time was used to calculate sprint speed. A 5-minute rest period was given between the two trials, and the fastest time was recorded for analysis.

### Genotyping

**Fig. 1:**
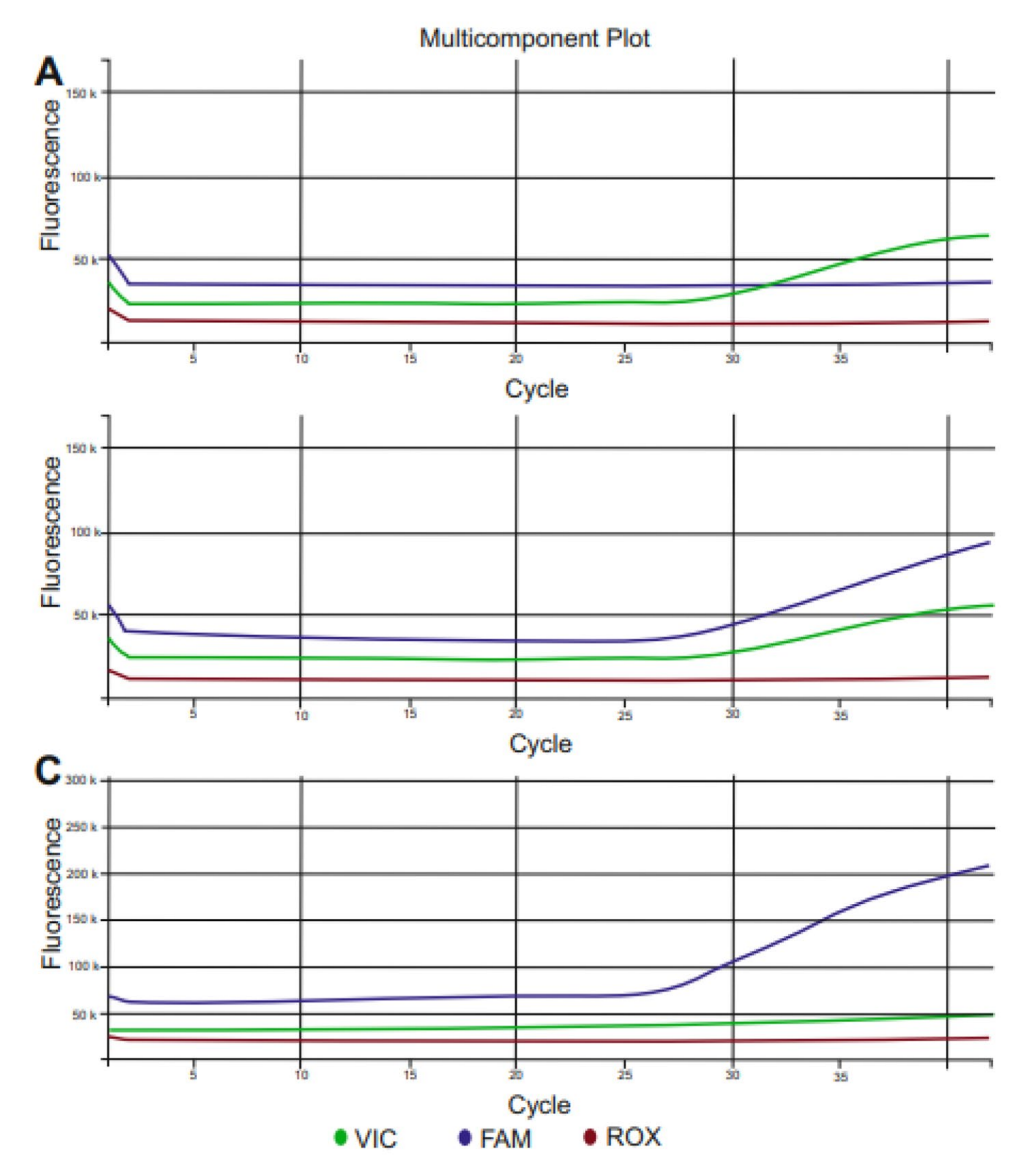
Multicomponent Plot images in Real-Time PCR of the CC, CT, and TT genotypes of the *ACTN3* rs1815739 polymorphism. The T allele (blue curve) is indicated by the FAM dye, while the C allele (green curve) is indicated by the VIC dye. **(A)** CC genotype is shown with a single green curve, **(B)** CT genotype is shown with both green and blue curves, and **(C)** TT genotype is shown with a single blue curve.

**Fig. 2:**
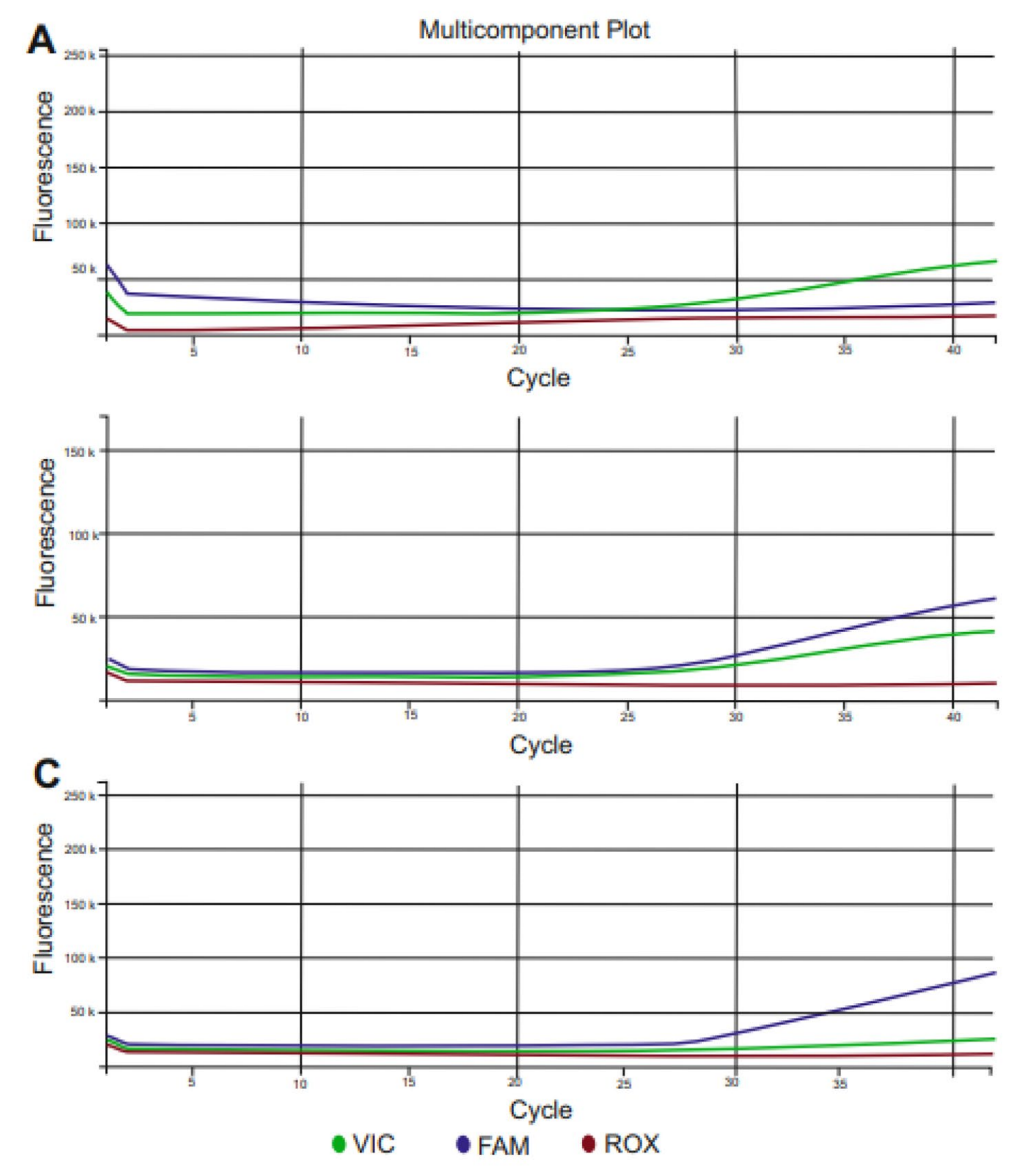
Multicomponent Plot images in Real-Time PCR of the CC, CG, and GG genotypes of the *PPARA* rs4253778 polymorphism. The G allele (blue curve) is indicated by the FAM dye, while the C allele (green curve) is indicated by the VIC dye. **(A)** CC genotype is shown with a single green curve, **(B)** CG genotype is shown with both green and blue curves, and **(C)** GG genotype is shown with a single blue curve.

Epithelial cell samples were collected by signing prospective forms from all participants. Working procedures were carried out according to the Helsinki Declaration II principles. DNA isolation was carried out by using a commercially available Canvax DNA Isolation Kit (Canvax Reagents S.L., C. Luis de Mercado, Boecillo, Valladolid, Spain). The operations were carried out with respect to the manufacturer’s instructions. All the genotyping procedures were carried out by Real-Time PCR (StepOne Plus, USA). For genotyping process, *ACTN3* rs1815739 and *PPARA-α* rs4253778 TaqMan SNP Genotyping Assays (Termofisher, USA) were used by following the manufacturer’s guide. Genotyping was completed by using 5 µL of master mix, 3.75 µL of H_2_O, 0.25 µL of the assay, and 1µL (10 ng) DNA, for a total of 10 µL [41]. The T allele for the FAM primer and the C allele for the VIC primer, used for the *ACTN3* TaqMan SNP Genotyping Assay, was identified (Table 2). The G allele for the FAM primer and the C allele for the VIC primer, used for the *PPARA-α* TaqMan SNP Genotyping Assay, was identified in Fig. 1. The TaqMan Probe sequences used for genotyping are shown (Fig. 2).

**Table 2:** Sequences of the TaqMan probe used for genotyping *ACTN3* rs1815739 and *PPARA* rs4253778 polymorphisms.

qPCR	Genes	Sequence, 5’-3’
VIC/FAM	*ACTN3*	CAAGGCAACACTGCCCGAGGCTGAC**[T/C]**GAGAGCGA GGTGCCATCATGGGCAT
	*PPARA*	ACACTTGAAGCTTGATATCTAGTTT**[G/C]**GATTCAAAA GCTTCATTTCCCATAT

### Statistical analysis

The statistical analysis of the data was conducted using the SPSS 25.0 computer program. Descriptive statistical methods (such as number, percentage, mean, and standard deviation) were used to evaluate the data. Based on the results of the Kolmogorov-Smirnov and Shapiro-Wilk tests, it was found that the data did not follow a normal distribution. As a result of these procedures, data from 16 soccer players with incomplete or incorrect information were excluded from the analysis, leaving data from 22 soccer players for further analysis. The Wilcoxon Signed Rank Test was used to examine the differences between pre-test and post-test performance of the soccer players. Hypotheses were tested with a 95% confidence interval and a significance level of p < 0.05.

## Results

**Fig. 3:**
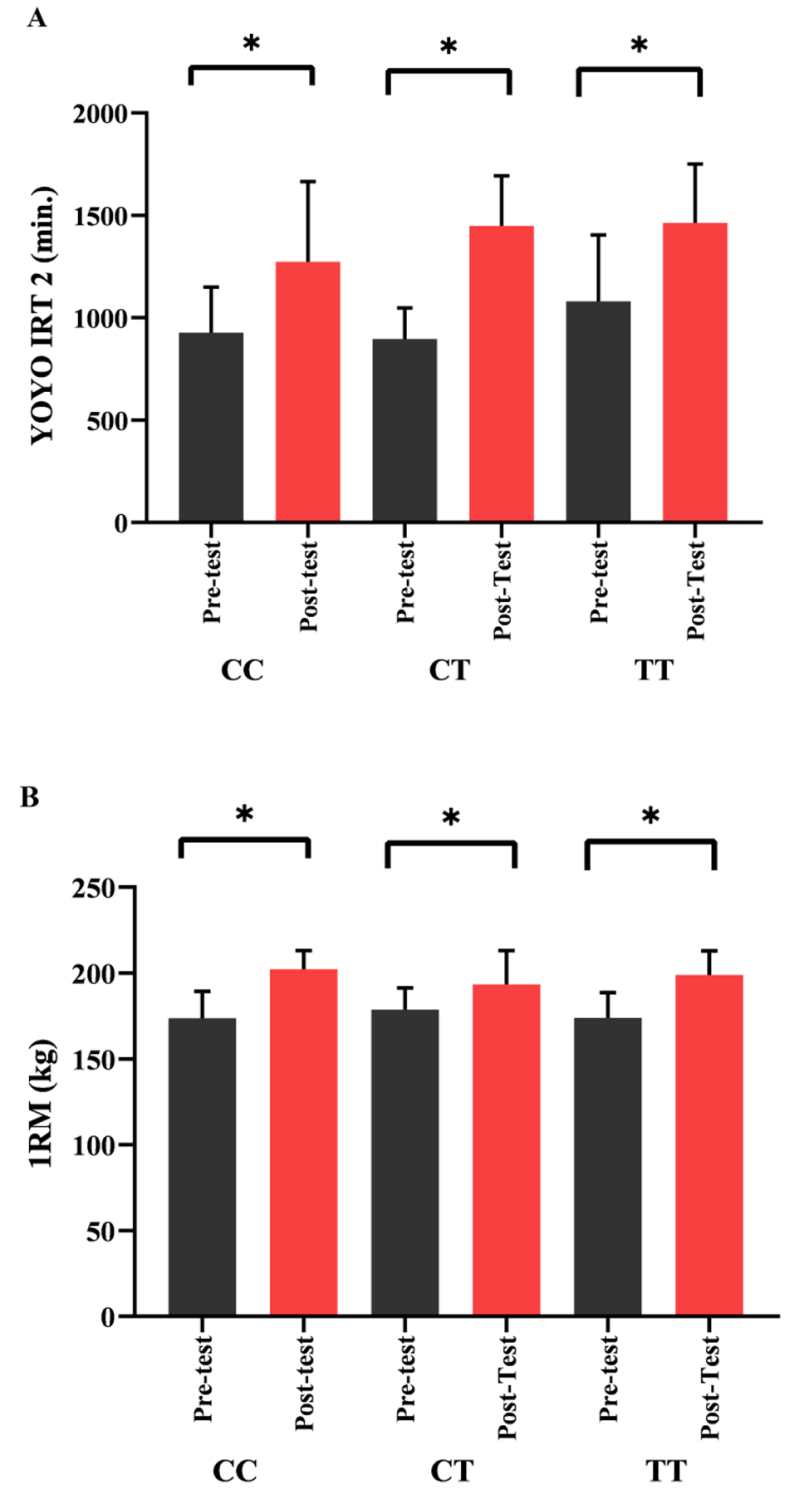
The relationship between **(A)** YO-YO IRT-2, **(B)** 1 RM and *ACTN3* rs1815739 gene polymorphism (CC, CT and TT), *p < 0.05

**Fig. 4:**
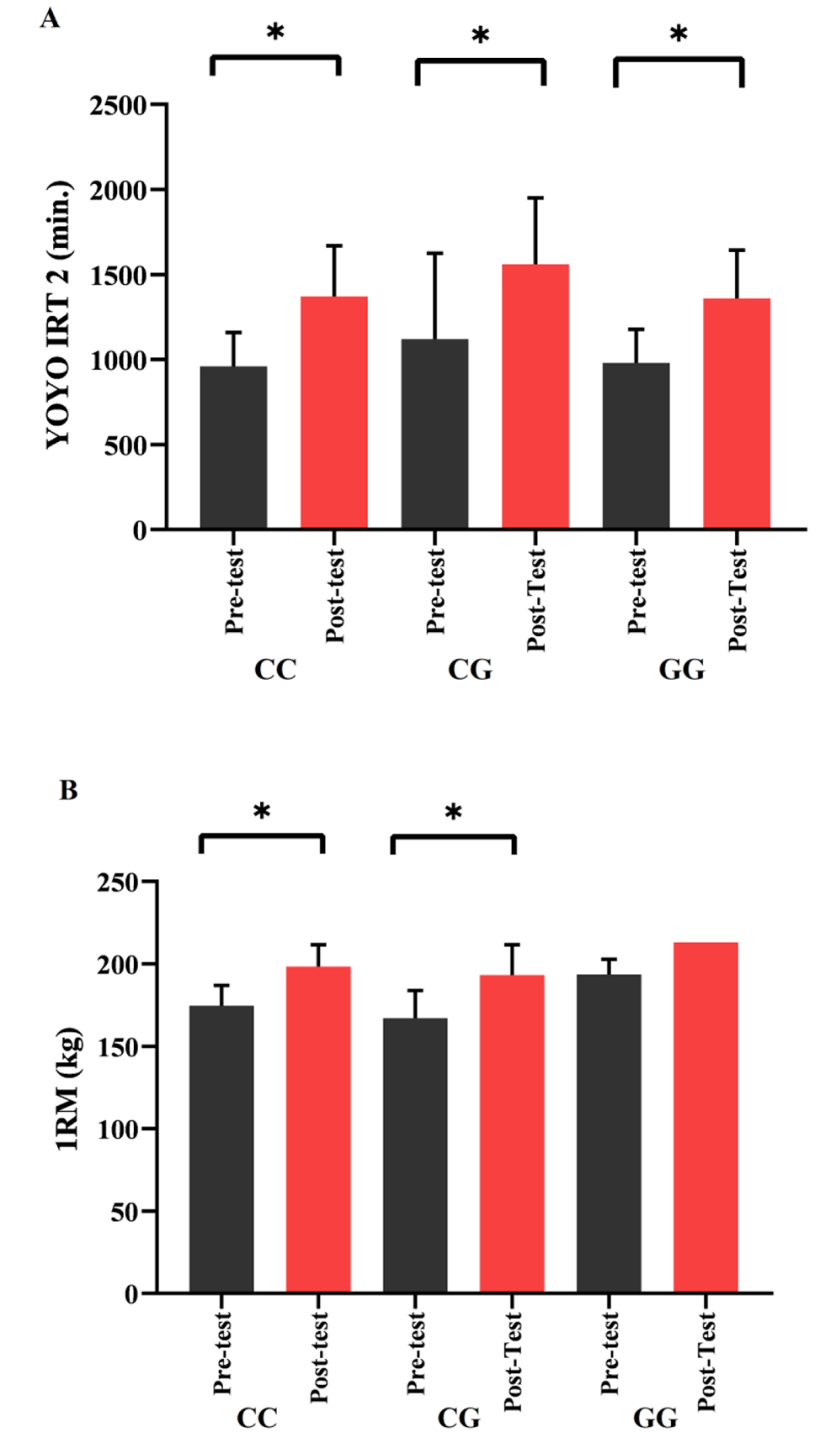
The relationship between **(A)** YO-YO IRT-2, **(B)** 1 RM and *PPARA-α* rs4253778 gene polymorphism (CC, CG and GG), *p < 0.05.

Table 3 shows the Wilcoxon results of the soccer players based on the *ACTN3* genotype variable, although no statistically significant difference was found in the SJ, 30m, CMJ, and DJ performance tests (*p* > 0.05), it was determined that there was a statistically significant difference in the YOYO IRT 2 and 1RM test results. (YOYO IRT 2: CC, CT, and TT *p* = 0.028, 0.003, 0.000; 1RM: CC, CT, and TT *p* = 0.010, 0.03, 0.001, respectively; Fig. 3). In Table 4, when the Wilcoxon results were examined based on the *PPARA-α* genotype variable, no statistically significant difference was found in the SJ, 30m, CMJ, and DJ performance tests (*p* > 0.05). However, there was a statistically significant difference in the YOYO IRT 2 and 1RM test results (YOYO IRT 2: CC, CG *p* = 0.001, 0.020; 1RM: CC *p* = 0.000; Fig. 4). The distribution frequencies of the *ACTN3* and *PPARA-α* genes were respectively, CC 28%, CT 28%, and TT 44% & CC 68%, CG 24%, GG 8%.

**Table 3:** The relationships between *ACTN3* rs1815739 gene polymorphism and athletic performance characteristics in professional soccer players

Variable	Genotype	n	Pre-test	Post-test	z	p
**SJ (cm)**	CC	7	37.48 ± 3.12	37.68 ± 3.22	-,423	.798
	CT	5	38.62 ± 2.65	37.26 ± 2.82	-1.214	.220
	TT	10	40.92 ± 5.18	41.36 ± 4.74	-1.172	.565
**YOYO IRT 2 (min)**	CC	7	926.66 ± 222.59	1273.33 ± 391.44	-2.201	.028*
	CT	5	896.00 ± 151.26	1448.00 ± 245.60	-2.032	.003*
	TT	10	1080.90 ± 323.38	1463.00 ± 287.75	-2.668	.000*
**30m (sec)**	CC	7	4.16 ± 0.05	4.13 ± 0.04	-1.187	.230
	CT	5	4.09 ± 0.09	4.08 ± 0.12	− .405	.692
	TT	10	4.07 ± .0.12	4.04 ± 0.11	-1.129	.256
**CMJ (cm)**	CC	7	40.12 ± 5.73	39.70 ± 4.60	− .338	.725
	CT	5	41.60 ± 3.96	42.95 ± 7.34	− .946	.401
	TT	10	40.01 ± 3.50	39.96 ± 4.14	− .204	.930
**DJ (sec)**	CC	7	38.94 ± 5.92	37.51 ± 4.44	-1.101	.300
	CT	5	40.24 ± 5.78	39.46 ± 7.31	− .944	.562
	TT	10	38.31 ± 2.80	39.05 ± 3.76	− .866	.348
**1RM (kg)**	CC	7	173.71 ± 15.59	202.28 ± 10.84	-2.371	.010*
	CT	5	178.60 ± 12.81	193.40 ± 19.65	-2.032	.034*
	TT	10	173.90 ± 14.78	198.90 ± 13.93	-2.803	.001*

*p < 0.05

**Table 4:** The relationships between *PPARA-α* rs4253778 gene polymorphism and athletic performance characteristics in professional soccer players

Variable	Genotype	n	Pre-test	Post-test	z	p
**SJ (cm)**	CC	16	38.35 ± 3.93	38.60 ± 3.73	− .597	.624
	CG	4	39.73 ± 4.60	39.61 ± 5.32	.000	.904
	GG	2	44.70 ± .42	42.75 ± 4.87	− .447	.647
**YOYO IRT 2 (min)**	CC	16	960.60 ± 197.58	1370.00 ± 298.01	-6.534	.001*
	CG	4	1120.00 ± 504.90	1560.00 ± 390.55	-4.554	.020*
	GG	2	980.00 ± 197.98	1360.±0.00	-6.333	.100
**30m (sec)**	CC	16	4.09 ± 0.11	4.07 ± 0.11	-1.604	.153
	CG	4	4.08 ± .0.07	4.13 ± 0.10	− .447	.126
	GG	2	4.08 ± .0.07	4.13 ± 0.10	-1.129	.605
**CMJ (cm)**	CC	16	40.65 ± 3.77	40.56 ± 4.43	.134	.895
	CG	4	40.14 ± 6.67	41.40 ± 8.57	− .855	.441
	GG	2	39.70 ± 1.55	39.55 ± 0.35	.111	.930
**DJ (sec)**	CC	16	39.68 ± 4.23	38.75 ± 4.18	1.402	.181
	CG	4	36.77 ± 6.46	37.02 ± 7.49	− .249	.820
	GG	2	37.40 ± 1.27	41.10 ± 4.52	-1.609	.354
**1RM (kg)**	CC	16	174.56 ± 12.35	198.31 ± 13.38	-5.848	.000*
	CG	4	167.00 ± 16.87	193.25 ± 18.37	-2.421	.094
	GG	2	193.50 ± 9.19	213.00 ± 0.00	-3.000	.205

*p < 0.05

## Discussion

The findings obtained from previous research indicate that the *ACTN3* and *PPARA-α* genes are highly effective candidate genes for physical performance development. In the current study, the individual and combined effects of *ACTN3* rs1815739 and *PPARA-α* rs4253778 gene polymorphisms on a targeted exercise program in professional soccer players have been evaluated. The study results, explaining that genetic inheritance is the source of both elite and non-elite athletic performance abilities, report that biomotor abilities (endurance, speed-power, and strength) are largely influenced by genetic structure [42–44]. Muscle strength and power parameters are influenced by multiple genes [45], and it is estimated that between 30% and 80% of these parameters are inherited. The study by Maciejewska et al. (2019) reported that the elite power athlete status was associated with at least 69 genetic markers [46]. Additionally, previous studies have shown that among these genes, 11 DNA polymorphisms that allow high levels of strength gain were identified, including the *ACTN3* and *PPARA-α* genes [47–49].

Many studies explain the positive impact of genetic variants on physical performance in professional or amateur soccer players [19], including speed, power [50], endurance [51], functional muscle strength, and power or agility [52]. Soccer is a sports discipline that requires both endurance and explosive power, such as long runs, jumps, sudden changes of direction, and sprints [53–56].

In some studies evaluating the effects of various genetic polymorphisms on speed, power, and strength performance in professional soccer players, positive relationships have been found between the *ACTN3* gene [55, 57] and "speed, power, and strength" genotypes [58–60]. Recent studies suggest that Olympic-level athletes who engage in strength sports need to have at least one copy of the "R" allele (alpha-actinin-3 protein production) of the *ACTN3* gene to achieve successful performance. Additionally, it has been proposed that XX genotypes have low levels of fast-twitch muscle fibers and testosterone. The aforementioned observations and studies indicate that the deficiency of alpha-actinin-3 limits the explosive force function required for speed running and power performance of fast-twitch muscle fibers [61]. Numerous studies have extensively discussed the R577X polymorphism within the *ACTN3* gene as a crucial determinant influencing the formation of various types of muscle fibers, thereby impacting an individual’s athletic performance. These studies have shown that elite sprinters or power athletes and healthy individuals with the RR genotype trigger more hypertrophy in the vastus lateralis muscles and have a higher proportion of fast-twitch muscle fibers [62, 63]. In addition to these, another study examining the relationship between the *ACTN3* rs1815739 polymorphism and physical performance in 138 Brazilian soccer players reported no significant genotype-phenotype association [64].

In previous studies, it has been reported that elite sprinters and athletes with a focus on speed, power, and strength have significantly higher frequencies of the R allele compared to controls [10, 65, 66]. In different studies, it has been reported that *ACTN3* RR genotypes, which are associated with elite athlete status, show better improvements in speed, power, and high-intensity resistance training compared to XX genotypes [12, 13, 67–69]. Moreover, it is believed that XX genotypes with alpha-actinin-3 deficiency may affect individual performance in sports such as power, sprint, soccer, and basketball [12, 19, 20, 70]. However, Alfred et al. (2011) have shown that European sprint/power athletes have a higher prevalence of the *ACTN3* R577X RR genotype compared to their non-athletic participants [71]. Ulucan et al. (2015) reported that the *ACTN3* RR genotype is more prevalent than the XX and RX genotypes, and similar findings have been observed in Russian (n = 240; 46.25% RR) and Brazilian (n = 60; 48.3% RR) soccer players [56, 58, 72]. In the literature, studies have associated *ACTN3* RR and RX genotypes with athletes who require high-intensity, explosive power in individual sports such as team sports and 100m sprint [12, 17], while the XX genotype is generally associated with aerobic endurance performance required for low-intensity, long-distance activities [24, 73]. Additionally, Yang and colleagues (2003) identified a higher frequency of the *ACTN3* RR genotype and R allele in registered professional athletes who require physical strength and power capacity in their activities [12].

Considering that soccer is a multifaceted sport that encompasses a combination of anaerobic, aerobic, and intermittent efforts during each match, the distribution of *ACTN3* genotype frequencies in soccer players appears to be more homogenous compared to other sports. As a result, soccer players may exhibit a more balanced distribution of *ACTN3* genotypes, accommodating the various physiological requirements of the sport. Similarly, other studies conducted in Brazil and Spain also failed to distinguish between professional soccer players and the general population [56]. Considering the potential benefits of the R allele for power and sprint performance and the X allele for endurance performance [74], it can be easily understood that both alleles (R and X) can play an important role in defining soccer and developing players who have important phenotypes in both strength and endurance. Similarly, the distribution of *ACTN3* polymorphisms among soccer players showed similarity in both genotypes. On the other hand, Yang et al. (2003) found that there was no correlation between higher XX genotype frequency and endurance performance in endurance athletes compared to controls. The same researchers showed that although Kenyan and Ethiopian endurance athletes had excellent success rates, the frequency of the XX genotype was observed to be quite low in these populations [12]. In addition, Gentil et al. (2011) reported that the R577X polymorphism in the *ACTN3* gene was not associated with resting muscle strength and the response of muscle strength to resistance exercise [75]. Similarly, no difference was found in *ACTN3* genotype frequencies between the fastest sprinters of all time and non-athlete controls (at least one R allele was detected in 97% of non-athlete controls) [76]. According to two studies conducted on resistance training, it was found that the RR genotype had an advantage in strength and power gain after resistance training [77, 78]. In contrast to these studies, it was reported that resistance training adaptations were not related to *ACTN3* gene polymorphisms [79]. Furthermore, Papadimitriou et al. (2016) reported in their study on elite sprinters that both a male and a female 100m sprinter who met the Olympic qualifying criteria did not have the R allele [4]. Another study that shows similarities with the current study findings (Table 3) reported that carriers of the X allele had greater gains in one-repetition maximum (1RM) strength compared to RR genotypes [80]. Similarly, in a study conducted by Garatachea et al. (2014) investigating the relationship between the *ACTN3* R577X polymorphism and explosive leg power in elite basketball players, no association was found between the *ACTN3* R577X polymorphism and the likelihood of being an elite basketball player when considering the results of squat jump (SJ) and countermovement jump (CMJ) tests [81]. The present study showed that no significant relationship was found between SJ and CMJ test results and *ACTN3* polymorphisms (Table 3). In another study conducted on healthy young adults (n = 283; 216 males and 67 females) and elite basketball players (n = 102; 61 males, 41 females), similar results were obtained, indicating that explosive power production capacity was not significantly affected by the *ACTN3* R577X polymorphism and that genotype frequencies were comparable between the basketball and control groups [82]. In addition, several studies [62, 80] have reported that the *ACTN3* R577X polymorphism had no effect on muscle strength and power parameters. Similarly, many studies [83, 84] support that there was no statistically significant association between *ACTN3* variation and the status of top athletes. In contrast to the above-mentioned studies, it has been noted that the observed gene frequencies of the *ACTN3* gene in professional soccer players (n = 40) were determined to be RX > RR > XX, and XX genotypes were observed at a relatively low frequency within the general population. These data suggest a trend that the *ACTN3* polymorphism may be an indicator of natural selection for sports that involve several biomotor characteristics such as speed, strength, power, endurance, and agility, such as soccer. In this study, it was observed that the R allele in males had a higher probability of being an elite soccer player in terms of performance compared to participants with the X allele. However, it was also stated that personal skills are of great importance in the soccer field. In the continuation, Coelho et al. (2016) remarked that factors affecting high performance in team sports such as soccer cannot be attributed solely to physical fitness but can also be influenced by the technical skills and tactical applications of each individual [42]. The findings suggest that *ACTN3* R577X is not an ideal genetic marker for identifying a talented soccer player. However, the results indicate that the likelihood of being a professional soccer player is higher for individuals with the *ACTN3* RX genotype compared to other genotype combinations [85]. In a study conducted by Massida et al. (2014) with soccer players and control groups, they expressed that no significant differences were found in the *ACTN3* genotype distributions (*p* > 0.05). This may be due to differences in anthropometric and biomechanical factors, as well as the presence of many other genes and environmental factors that determine physical performance along with fitness levels [54].

In literature, previous studies showed that there were differences between *ACTN3* variants [53, 86, 87]. It is known that jumping plays a crucial role in achieving superior performance in soccer games, and a high correlation has been found between jumping height and sprint performance. Contrary to the our findings, Pimenta et al. (n = 200) found that among elite soccer players, those with RR and RX genotypes had higher scores in countermovement jump (CMJ) and squat jump (SJ) tests (RR 38 cm, RX 37 cm, XX 35 cm) compared to XX genotyped players (*p* < 0.05) [55]. However, different researchers have not found any relationship between jump and 10-20-30 m sprint tests and genotype distribution among athletes playing in professional, U17, and U20 age groups [42, 88], which is consistent with our findings. Likewise, Pimenta et al. (2013) found that in studies conducted on soccer players, the VO2max measurement values determined by the Yo-Yo INT 2 test were higher in XX-genotyped players than in RR-genotyped players. Additionally, no difference was found in VO2max measurements determined by the Yo-Yo INT 2 test according to *ACTN3* genotype, and it was observed that RR-genotyped soccer players had faster and higher jumping potential in short-distance runs [55]. In the present study, no significant results were found in the sprint (30m) and jump tests (SJ, DJ, CMJ) measurement results of *ACTN3* genotypes, but significant results were obtained in YOYO INT 2 test results, which were determined as TT > CC > CT genotypes, respectively (Table 3). Although soccer is considered a long-term exercise, it is well known that matches involve high-intensity short-term efforts (sprints or jumps). Therefore, in elite soccer matches, in addition to technical and tactical skills, muscle strength and “explosive” leg power are the most important factors contributing to successful performance [55]. However, it is stated that the main reason for the success of teams competing in the top of the Italian and English Premier Leagues is not the intensity of high-intensity efforts, but rather the better technical and tactical efficiency [89, 90]. On the other hand, a strong correlation has been described by many studies between the higher frequency of the R allele and power and speed athletes (weightlifting, sprinting, short-distance swimming, etc.). For instance; Yang et al. (2003) reported that none of the speed-power athletes observed at the Olympic Games were carriers of the XX genotype, and carriers of the R allele showed higher muscle strength, power, and speed [12]. Furthermore, previous studies have reported that R allele carriers achieved higher maximum strength and more muscle mass after a 9-week lower limb resistance training [62, 88, 91]. Similarly, Petr et al. (2022) evaluated the effects of *ACTN3* variants in a group of elite soccer players and found that in terms of quadriceps and hamstring isokinetic strength and jump performance (at speeds of 60°/s, 180°/s, and 300°/s), defensive players with XX alleles had lower quadriceps and hamstring isokinetic strength than RX and RR genotypes at all tested speeds. In RR genotype defensive players, however, it was observed that they had higher quadriceps muscle strength than RX genotypes at all three speed levels. Furthermore, it was stated that strength performance was higher in attacking and defensive players with the R allele compared to midfielders, and midfielders had lower strength and power capacity compared to other playing position [92]. Soccer is a sport that combines the parameters of strength and speed. Although aerobic energy systems are perceived as the most required energy source for soccer, the main source of the short-term, high-intensity attacks that are competitive and intense in soccer games is ATP-PC and anaerobic glycolysis (anaerobic energy systems) [93]. During the match, high-speed sprints make up approximately one-eighth of the total distance covered [32], while tactical applications can change the intensity of high-intensity loads in the game [89, 94]. However, it is known that the number of high-intensity sprints during the match can provide an advantage for the team, but it is not possible to apply this sprint performance in every game. The reason for this could be related to changes in the players’ physical condition during the season, as well as the intensity, density, and displacement factors of the training loads applied, which may be related to the displacement factors caused by traveling [35, 95, 96].

Contrary to our study findings (Table 3), in a study comparing the performance capacities of different strength, speed, and endurance tests of *ACTN3* gene variants in players (n = 200) of the Super League, it was found that individuals with RR genotype completed a 30- meter distance in a shorter time compared to RX and XX individuals. Similarly, it was reported that individuals with the RR genotype completed jump tests in a shorter time compared to individuals with the RX and XX genotypes, while individuals with the XX genotype showed higher maxVO2 values in aerobic tests compared to the RR group (p < 0.05). The findings of this study suggested that *ACTN3*/RR genotype soccer players had a higher potential for faster short distances and higher jumping ability, while *ACTN3*/XX individuals exhibited the highest aerobic capacity values [55].

In a study conducted by Eken et al. (2021) on 21 professional soccer players, the distribution of *ACTN3* gene polymorphism was found to be 28.6% CC, 38.1% CT, and 33.3% TT [93]. The studies including swimming, wrestling and professional soccer players found that the distribution rate of CC genotypes was higher compared to other genotypes [97]. In another meta-analysis study that found similar results, the *ACTN3* CC genotype was associated with strength-related phenotypes in soccer players, and strength-related phenotypes were reported to be important for success in soccer [43]. In contrast, the present study (Table 3) found that the TT polymorphism had the highest recorded success rates (CC 28%, CT 28%, and TT 44%).

In another study that investigated *PPARA-α* gene polymorphisms, the acute super-compensation effects of instant shuttle runs were evaluated using the Optojump device and 10-second continuous vertical jump tests in an inactive control group, elite endurance runners, and soccer players. It was found that the *PPARA-α* GC and GG alleles had a more advantageous effect on the level of sudden recovery compared to other alleles, and there was a significant combined effect of multiple genes on sudden super-compensation [98]. In contrast, the current study findings did not observe any differences in *PPARA-α* gene polymorphisms among SJ, DJ, and CMJ test results (Table 4). Specifically, research in endurance sports has indicated that the *PPARA-α* gene G allele, which uses oxygen more efficiently in conjunction with a high proportion of slow-twitch type I muscle fibers, plays an active role in the adaptation process of aerobic endurance training [21, 23, 24, 26]. According to our findings, it was determined that the C allele of *PPARA* gene in CC genotype soccer players had a more advantageous effect on their recovery levels (YOYO IRT 2) compared to other allele groups (GG, GC). Similar findings were also observed in 1RM test results (CC > CG > GG) (Table 4). Nevertheless, it was found that the C allele of the *PPARA-α* gene was particularly effective in anaerobic activities where the proportion of type II muscle fibers was higher, especially in trained athletes [23, 28, 58–60].

In previous studies with analogous findings, it was noticed that the frequency of the C allele was more prevalent among sprinters and athletes emphasizing speed and strength-oriented disciplines [59]. Conversely, the frequency of the G allele was found to be higher in endurance athletes [21]. Similarly, a different study has explained that in the case of the C allele, it was better adapted to training loads for strength abilities [99]. Many studies found a correlation between carriers of the C allele and higher levels of anaerobic performance, better WT30 test parameters, and higher muscle mass and strength [23, 28]. In fact, it was reported that the combined effects of individual genotypes R *ACTN3* and C *PPARA-α* on counter-movement jump test parameters were statistically significant. In contrast to the current study, it was observed that the combination of X *ACTN3* and C *PPARA-α* genotypes was significant [98, 100]. In another study, it was stated that athletes with *vA* CC and CG genotypes had more muscle mass and higher vertical jump scores compared to athletes with GG genotype characteristics. Lastly, it was observed that the GG genotype was more prevalent in endurance athletes as compared to sprinter athletes [101].

Alvarez-Romero and colleagues (2020) indicated that the C allele of the *PPARA-α* gene represented a significant advantage for the trainability of strength abilities [99]. However, it was found that CC genotypes expanded three times and GC alleles expanded two times, which was associated with left ventricle enlargement as an adaptation to external physical load [102]. In the present study, as observed in the YOYO INT 2 test results, the presence of the C allele in trained individuals indicated a possible advantage in terms of anaerobic metabolism [23]. In another similar study conducted by Meckel et al. (2019), it was found that young soccer players had a lower percentage of *PPARA-α* CC genotype rate (15%) compared to long-distance runners (19%) but had a higher CC rate (10%) in sprints and jumps [103].

Soccer is a high-intensity activity that requires a combination of speed, strength, and power parameters such as short sprints, fast attacks, jumps, and zigzag runs to achieve game dominance along with prolonged physical loads executed at moderate to low intensity. Individual abilities of players, technical and tactical applications, motivation, and fitness level are among the most important factors that affect the outcome, along with differences in performance resulting from interpersonal attraction in both competitive matches and training. Considering the fact that genetic characteristics affect the degree of adaptation to training, planning personalized training programs for individuals is thought to contribute to the improvement of both individual and team performance as well as the efficiency of in-game performance. Candidate genes that reveal athletic ability, when used in elite athletes performing at a high level, can provide important clues for the scope and intensity of training practices, loading, rest and recovery processes, and the emergence of in-game high-level performance. The key is whether the candidate genes studied can determine natural advantages or abilities as part of training practices. Undoubtedly, athletes who interact with the correct methods and practices will have a higher likelihood of achieving higher levels of performance [1, 14, 28, 29].

The present study acknowledges certain limitations that need to be taken into account when interpreting its findings. Firstly, the sample size is relatively small, potentially impacting the precision of the results and the significance of observed differences. A larger sample size could lead to more robust conclusions. Secondly, the focus on only two candidate genes in training adaptation might influence the evaluations in a biased manner. Considering additional genetic factors and their interactions could provide a more comprehensive understanding of the genetic reflections on athletic performance. Furthermore, it is essential to consider the homogeneity and quantity of the study groups when analyzing the relationship between genotype and athletic performance. These variables play a crucial role in ensuring the reliability and consistency of genetic associations with athletic attributes. To enhance the reliability and generalizability of the findings, future research efforts could integrate biochemical data from larger and more diverse groups across various sports disciplines. This approach would provide a more comprehensive picture of the genetic factors that impact athletic performance and contribute to the advancement of sports science.

## Conclusion

The present study unveiled significant improvements exclusively in the YOYO INT 2 and 1RM test results across the majority of gene variants following the six-day-a-week training program. Other performance tests, such as the SJ, 30m sprint, CMJ, and DJ Test, did not show statistically significant differences. The findings suggest that the genetic variations studied are more closely associated with improvements in endurance (YOYO INT 2) and maximal strength (1RM) aspects of athletic performance rather than explosive power and speed-based measures. Indeed, the results of our study suggest that these tests, particularly the YOYO INT 2 and 1RM assessments, can serve as alternative tools to evaluate the athletic performance of individuals with specific genetic polymorphisms (*PPARA-α *rs4253778 and *ACTN3* rs1815739) that were examined in our research. By considering the genetic variations identified in our study, coaches, trainers, and sports scientists can use these performance tests as valuable indicators to assess the potential strengths and weaknesses of athletes in specific areas, such as endurance (YOYO INT 2) and maximal strength (1RM). These alternative assessments offer insights into the athletes’ abilities that may align with their genetic predispositions, enabling tailored training and performance enhancement strategies based on individual genetic profiles. Integrating genetic information into the evaluation process can further optimize training programs and aid in talent identification, ultimately contributing to the development of personalized training regimes for athletes, thus maximizing their athletic potential. Moreover, these results highlight the importance of considering specific genetic influences on different aspects of athletic performance to gain a more comprehensive understanding of the molecular processes underlying sports-related traits. Given this context, it is evident that more extensive research is essential to enhance the predictability of results, considering the potential influence of two candidate gene polymorphisms eliciting diverse characteristics that may positively affect the on-field performance of soccer players.

## Data Availability

The datasets presented in this study can be found in online repositories. The names of the repository/repositories and accession number(s) can be found at: https://figshare.com/, 10.6084/m9.figshare.23684466.v1
